# The Who or the How? Species vs. Ecosystem Function Priorities in Conservation Ecology

**DOI:** 10.3389/fpls.2021.758413

**Published:** 2021-11-02

**Authors:** Sebastian Leuzinger, Boris Rewald

**Affiliations:** ^1^School of Science, Auckland University of Technology, Auckland, New Zealand; ^2^Department of Forest and Soil Sciences, University of Natural Resources and Life Sciences (BOKU), Vienna, Austria

**Keywords:** conservation ecology, ecosystem function and ecosystem services, exotic species, functional diversity, functional traits, invasion impact, invasive species, plant invasion

## Abstract

Current conservation strategies are targeted at preserving species, without explicitly aiming at the maintenance of ecosystem functions. In a physically highly connected world, the unintentional relocation of terrestrial, marine, and microbial life is therefore unavoidable and has been an integral part of human evolution for thousands of years. Here, we challenge the default perception often shared among conservation ecologists that preserving native species at all costs and reducing the number of exotic species and their abundance is the only way to conservation and restoration success. While this strategy is valuable in cases where exotic species disrupt ecological function, there are examples where exotic species have similar functional traits to the threatened or extinct native species and can in fact help maintain the overall or target function of an ecosystem. In the race to cope with global environmental change, we argue that ecosystem function and ecosystem services need to be viewed not only through a taxonomic lens, but increasingly also through a functional, trait-based one.

## Introduction

The definition of biological species ultimately rests on human-made concepts, particularly in the realm of unicellular organisms (Hanage et al., 2005). Conversely, biological function (or traits) can more objectively be measured. For example, the rise of oxygen in the early atmosphere of the earth was caused by bacteria and had a massive and irreversible impact on all subsequent life. The quality and size of the impact, however, depended on the organisms’ traits, i.e., it was entirely of *functional* nature, and did not hinge on what species and how many were involved – the *how* mattered, not the *who*. Traits are features of an individual organism that potentially affect the performance or fitness of the organism itself (“response traits”). However, a particular trait, or set of traits, of an organism also impacts its biotic and abiotic environment (“effect traits”; Díaz et al., 2013). Plant traits can be physical/morphological (e.g., leaf size and thickness), biochemical/physiological (secondary metabolite production, leaf stoichiometry, carbon assimilation pathway), or temporal/phenological (timing of leaf-out, leaf shedding; Violle et al., 2007). Great advances have been made in establishing global, publicly available trait data (Kattge et al., 2011), although severe gaps in the availability of functionally “relevant” traits, and their intraspecific variability and plasticity under various environmental conditions, persist (Freschet et al., 2021). Importantly, a given trait (or function) is not necessarily associated with a particular species but can be similar in/performed by different taxa (Calow, 1987), although the likelihood of two species showing similarities in multiple traits naturally decreases.

Since the establishment of the section *Functional Plant Ecology* within Frontiers in Plant Science 10-years-ago (Körner, 2011), this journal has been able to highlight many trait-based studies, lastly, e.g., on how functional traits can be used to predict species assemblages (Li et al., 2021). Notwithstanding that our comprehension of trait–function relationships is still evolving, particularly below ground (Bergmann et al., 2020; van der Plas et al., 2020; Freschet et al., 2021), there is a growing consensus that a trait-based approach has a strong potential to help us understand (1) how functions are coordinated within organisms (Kurze et al., 2021), (2) how species perform under varying environmental conditions (Nikolova et al., 2020), and (3) how species affect ecosystem functioning including the services delivered to humans (Liu et al., 2021).

The numbers of non-native species in floras are steadily increasing in Europe (Lambdon et al., 2008), the United States (Pimentel et al., 2005), but also on much more easily to protect islands like New Zealand (Hulme, 2020). Non-native (exotic) plants in general and alien (invasive) species in particular are thus a pervasive global challenge (Millennium Ecosystem Assessment, 2005) – particularly affecting conservation of biodiversity and ecosystem functioning. While “invasiveness” is naturally often based on measures of population growth and spread in the new region (Pyšek et al., 2004), defining invasiveness also by the impact on the invaded ecosystems has been suggested (Davis and Thompson, 2001). While non-natives can have both positive and negative impacts on their host ecosystems, positive effects are rarely reported (Simberloff et al., 2013; Blackburn et al., 2014; Sladonja et al., 2015). In this perspective piece, we raise the delicate question of whether ecological function needs to be more carefully weighed off against the sheer conservation of native species assemblages. This may sometimes mean the acceptance of the role of exotic species in performing similar ecological function(s) to that of natives, whose protection often involves an extremely high cost (Fairburn et al., 2004; Moore et al., 2011). Ultimately, and particularly in the light of global environmental change, the maintenance of ecological function, and thus ecosystem services, are key and indisputably more valuable than sheer biodiversity metrics. We argue that, 10 years after a debate weighing off the problem of non-natives vs. the broader anthropogenic impact on our planet (Hulme et al., 2011; Thompson and Davis, 2011; van Kleunen et al., 2011), an increasing understanding of response and effect traits of both invasive and native species is urgently needed to support efficient decision making in conservation ecology. While we focus on plants, we also borrow from faunal examples, as in the present question, the same principles apply to all organisms.

## Ecosystem Functioning as Dependent on Species and/or Functional Diversity

Ecosystem functions are the biotic and abiotic processes within an ecosystem. They are the foundation of ecosystem services (Costanza et al., 1997). Ecosystems are often managed or valued for several ecosystem functions – so-called ecosystem “multifunctionality” (Sanderson et al., 2004). However, sometimes particular functions are more important than others, e.g., in protection forests, mitigating or preventing the impact of rockfalls and landslides will be key (Moos et al., 2019), while, e.g., carbon sequestration will be of secondary importance. Under rapid environmental change, the key question is when and where such services rely on taxonomic vs. functional diversity.

**FIGURE 1:**
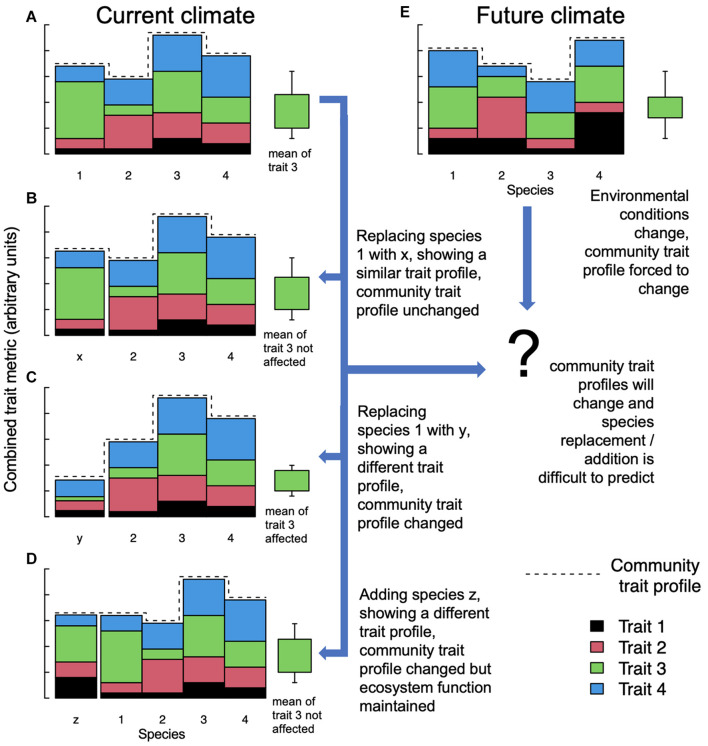
Model ecosystem with four species and four functional traits in current and future climates. Traits are stacked and combined to represent a combined trait metric (no units). The dashed line symbolizes the community trait profile, i.e., the specific set of functional characteristics that have evolved in response to a given environment (Russell et al., 2014). The green boxplot exemplifies the community-weighed mean and range of trait “3” under different scenarios. **(A)** A pristine ecosystem holding four species. In example **(B)**, species 1 is replaced by alien species x, which shares a similar trait profile to 1, such that neither the mean of trait 3 nor the community trait profile is affected. In **(C)**, alien species y replaces species 1, but because its trait profile is substantially different, both the mean of trait 3 across all species and the community trait profile are affected, which might be compromising overall ecosystem functioning. In **(D)**, species z is added to the community without replacing a species and without affecting overall ecosystem function under the current climate but potentially adding redundancy under future environmental conditions. Under future climate **(E)**, the community trait profile and parameter values of specific traits may change even if species assemblages are initially preserved, and thus the impacts of alien species x, y, and z on ecosystem services are much more difficult to predict and can be either positive, neutral, or negative.

The idea that species diversity *per se* could be an important determinant of ecosystem function (biodiversity-ecosystem functioning, BEF) has been debated for decades. For example, Isbell et al. (2011) show that if larger spatiotemporal scales are considered, functional redundancy is required *via* higher than expected species numbers. Similarly, Hector and Bagchi (2007) concluded earlier that because species often facilitate functions performed by others, studies focusing on individual processes in isolation will generally underestimate levels of biodiversity required to maintain multifunctional ecosystems. However, early studies addressing BEF were frequently criticized for not sufficiently separating complementarity (i.e., high-diversity plant communities can utilize resources more completely) from sampling (i.e., biased toward including highly productive or N-fixing species) effects (Eisenhauer et al., 2016). This resulted in experiments focusing less on taxonomic diversity but more on functional dissimilarity (Díaz and Cabido, 2001). As traits were shown to determine the contributions of species to ecosystem functions (Garnier et al., 2004; Funk et al., 2017), ecologists now often quantify trait variation within a species assemblage, generically referred to as “functional diversity.” Functional diversity thus presupposes a mechanistic link between diversity and ecosystem function (Cadotte et al., 2011). Additional drivers such as intraspecific variation, species interactions under contemporary evolution, and interwoven abiotic factors may be needed to improve predictions of ecosystem functioning by models (Carroll et al., 2007; Valiente-Banuet et al., 2015; Funk et al., 2017; van der Plas et al., 2020). As the concept of functional diversity is thus by definition removed from the notion of individual taxonomic species, a specific ecological function can be achieved by the trait profile of taxonomically entirely different species – so-called “functional homologs” (Love, 2007). Ultimately, however, potential changes in community-weighted means and trait ranges (i.e., the “community trait profile”; change in trait profiles and trait “3” are exemplified in Figure 1) re-shape the functioning and resilience of the colonized ecosystem (Russell et al., 2014; Sodhi et al., 2019). Addressing realistic, real-world conservations tasks, it remains thus open to debate if the contribution of exotic species – acting as homologs for natives and sustaining target ecosystem services – should not be considered in conservation and restoration decisions rather than maintaining species assemblages *per se*.

## Can Exotic Species Maintain Ecosystem Functioning, Ecosystem Services?

The movement and exchange of plants between people and regions have been one of the defining characteristics of the human species (Heywood, 2012). Apart from agricultural crops, the cultivation of non-native tree species for production (timber, industrial wood, and biomass) or restoration purposes is probably one of the most widely accepted uses of exotic species to promote specific ecosystem functions (Dodet and Collet, 2012). This is despite some of the introduced non-native species in Europe, e.g., *Acacia* spp. and *Robinia pseudoacacia* turned out to be invasive or induce unexpected disservices (Souza-Alonso et al., 2017; Šibíková et al., 2019; Langmaier and Lapin, 2020). However, *R. pseudoacacia*, for example, is simultaneously regarded as valuable for the restoration of degraded croplands (Papaioannou et al., 2016). Similarly, exotic pine plantations in New Zealand can support a diverse native flora in their understorey (Forbes et al., 2019). However, as monospecific plantations typically provide less non-production-related ecosystem services on all but least degraded sites (Bauhus et al., 2010), future planted forests may be designed, and management measures adapted, to integrate ineradicable alien species – maximizing multifunctionality (Liu et al., 2018; Messier et al., 2021). In specific, Baeten et al. (2019) reported that targeting high tree productivity does not necessarily trade-off against other ecosystem services. Thus, high productivity and multifunctionality can be combined with an informed management of tree species and assemblages that favors (target) ecosystem functions – combining native, locally dominant species with economically important exotics as well as locally present but ineradicable aliens alike. In urban forestry, where ecological function almost always outweighs the importance of species identity, the use of non-native species has become commonplace (Sjöman et al., 2016; Conway et al., 2019; Arrington, 2021).

Lastly, “assisted colonization” or “re-wilding,” almost exclusively used for animals and while heavily debated (Ricciardi and Simberloff, 2009; Schlaepfer et al., 2009), attempts to replace extinct or struggling natives with an alien homolog. This can work if the trait profile of the introduced alien is reasonably similar to the extinct species, and does not affect the overall community trait profile of an ecosystem (Figure 1B); this may of course change under altered environments (Figure 1E). One successful example is exotic tortoises that were released on Round Island, Mauritius to replace an extinct native tortoise. The functional trait profile of the introduced species seemed to match that of the extinct species sufficiently to restore overall ecosystem functioning (Griffiths et al., 2013). While “assisted colonization” often fails because of problems with the introduced species itself (see Fischer and Lindenmayer, 2000 for faunal examples), this threshold is removed in cases where aliens have already established, and their functional contribution to the ecosystem remains to be assessed. In the best case, such aliens complement the present species portfolio adding functional redundancy (Isbell et al., 2011; Figure 1D) or even serve as a replacement for lost natives as illustrated in the above example (Figure 1B).

More or less accidental or historic species introductions are ubiquitous and generally irreversible on a global scale, and if the community trait profile of the resulting ecosystem is sufficiently altered (Figure 1C) their impacts on ecosystems can be extremely detrimental (Lowe et al., 2000; Simberloff et al., 2013). In some cases, however, even though whole landscapes are transformed and species assemblages change, the overall function of the ecosystem remains remarkably unchanged. One example is the introduction of large succulents in the Mediterranean, which added a functional type rather than replacing one (Figure 1D; Vilà et al., 2003; Heywood, 2012). Often, if ecological function alone is considered, exotic species appear in a different light. The invasive tree species *Ailanthus altissima* (tree of heaven) increasingly occupies disturbed sites in Europe including forests in the Alps and is considered one of the worst invasive plants in Europe – not least due to its homogenization effects on species composition and impact on regeneration of (previously) dominating tree species (Sladonja et al., 2015; Langmaier and Lapin, 2020). *A. altissima* is thus the center of a large range of activities aiming to control the invasion, but eradication is difficult, as it has a high regenerative capacity (Sladonja et al., 2015). While stand conversion into high forests has been proposed as a potential way of controlling the invasion of light-demanding *A. altissima* (Radtke et al., 2013), this conflicts with the superiority of coppice stands in providing rockfall protection (Jancke et al., 2009). Indeed, a key functional trait of mountain forests is the protective capacity against rockfall, avalanche, and erosion, and alien *A. altissima* intruded forests in Southern Switzerland seem to be providing this function (and ecosystem service) similar to other tree species in the region (Moos et al., 2019). One of those species showing a similar decay pattern, and thus a similar potential decrease in energy reduction capacity against rockfall, is *Castanea sativa* (European chestnut). Ironically, *C. sativa* was originally also introduced to the area (∼2k years ago; Conedera et al., 2004), and now enjoys considerable conservation efforts to protect it (Pereira-Lorenzo et al., 2020). It is an interesting phenomenon how with prolonged exposure, humans seem to legitimatize “naturalized” alien species, sometimes through the association of a historic connotation. From a purely functional perspective, however, a newly introduced species should be assessed using the same criteria as species we may perceive as less “alien” simply because they have been around for longer.

## Discussion: Functional Diversity/Trait-Guided Decision Making in Ecosystem Management and Conservation

**FIGURE 2:**
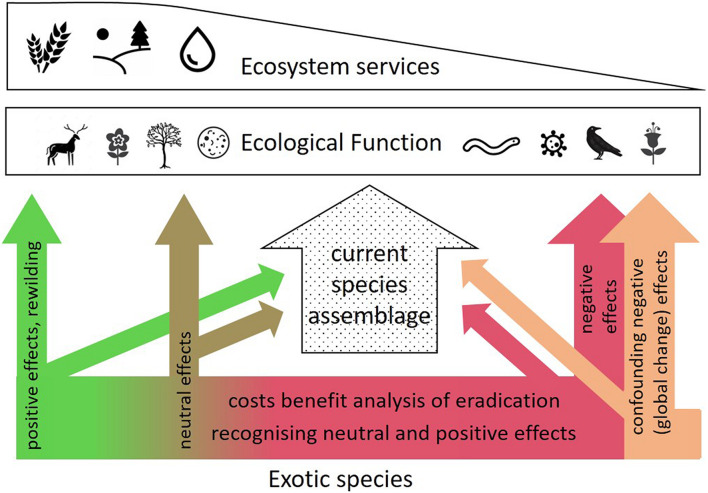
Schematic showing the role of exotic species in an ecological context. In a given location, the current species assemblage (center) supports ecological function and ultimately ecosystem services. The influence of exotics both on the current species assemblage and directly on ecological function can be negative (right-hand side, in red, termed invasive or alien species), neutral, or positive, the latter one *via* accidental or deliberate introductions (re-wilding). Negative effects are often overlaid by (and thus confounded with) other global change effects (orange arrow on the right). A careful analysis of exotic species that involves identification of potentially neutral or positive effects, and a cost/benefit analysis of detrimental invasives, is critical where resources for efforts are limited.

According to the simple rule “never change a running system” and without an exhaustive understanding of ecosystems, it is often argued that all species should be preserved in their natural habitat because one cannot be certain exactly which species provides which ecological function (Ehrlich and Ehrlich, 1981). In a fully globalized economy, however, species introductions are ubiquitous and irreversible (Sladonja et al., 2015), and their management should carefully consider both detrimental and beneficial effects both on biodiversity *per se* and purely functional aspects of an ecosystem (Figure 2). Detrimental effects on natives are further often confounded with other global change drivers including climate-driven range shifts (e.g., Johnson et al., 2011; Wallingford et al., 2020). Clearly distinguishing between the two effects that often act in parallel, is often difficult (Figure 2). This was also recognized in very early studies from New Zealand. Allan (1936) points out that the detrimental effects on native ecosystems come from the direct human interventions, and much less *via* invasive species themselves. Despite extremely strict biosecurity regulations and ample pest eradication programs (Goldson et al., 2015), the island has more invasive plant species than any other island (Hulme, 2020). At the same time, protecting rare natives/eradicate invasive species involves a high cost (Fairburn et al., 2004; Moore et al., 2011). Conversely, some invasive species may partly play a positive role, e.g., the European legume *Ulex europaeus* (gorse), which acts as a nurse plant for native forest regeneration in many areas of New Zealand, although plant succession under *U. europaeus* follows initially a different trajectory from that occurring under the homolog native species (Norton, 2009). These examples highlight the delicate trade-off between pure species conservation and a more functional (traits) approach.

The examples in Figure 1 illustrate how using trait-based frameworks might lead to better understanding and prediction of invasion impacts (see also Sodhi et al., 2019). We fully acknowledge that an incomplete understanding of hard-to-predict ecological interaction imposed by exotic species may pose a risk to multiple ecosystem functions. Examples of alien species disrupting local ecosystems disastrously are plentiful [e.g., Phillips et al. (2007), but see Lowe et al. (2000) for a complete list], and predicting such impacts is difficult (Dehlin et al., 2008). Efforts to avoid further unintentional introductions and dispersion must therefore always be supported, particularly where this can be achieved at a reasonable cost. However, the broad implementation of risk assessment strategies based on functional traits, facilitating the prediction of the capacity of a species to affect ecosystem functions, and to maintain or enhance these functions under future environments (Díaz et al., 2013; Cuthbert et al., 2019) may help inform a more nuanced ecosystem management approach. This is particularly true where neither exclusion nor eradication is realistic, or in cases where alien species either have a neutral or positive effect on BEF (left arrows in Figure 2). It is fully acknowledged that our understanding of trait-function relationships, particularly including the consequences of variation and plasticity of trait sets under extreme climatic conditions and a rapidly changing environment, must evolve further to increase prediction accuracies (Figure 1, center right).

In brief, and colloquially expressed, it comes down to the question to what extent we can afford “open-air museums,” and when it is more cost-effective to maintain ecosystem function, i.e., *how* (well) is the job done, not by *whom*. Ultimately, this may result in classification schemes for alien species beyond the current focus on the adverse impacts (e.g., Blackburn et al., 2014). Also, as Brodie et al. (2018) suggest, such schemes must look beyond the survival of individual species, but target key roles in species interactions and the maintenance of communities and ecosystems. In any case, there are valid arguments beyond ecological function [e.g., aesthetic, ethical, or cultural (Lindemann-Matthies et al., 2010; Aerts et al., 2016; Sacchelli et al., 2020)], which justify the combat of invasives and/or the protection of natives in their own right. In particular, societal and political processes may set the normative values guiding management decisions.

However, the partially positive impact of exotic and even invasive species on the functional diversity of species assemblages and target ecosystem functions, as exemplified above, seem currently not considered sufficiently in real-world ecological management decisions. We therefore strongly advocate for an evidence- and function-based decision-making process beyond conserving species assemblages *per se* (see Figure 2 for illustration). To evaluate to which extent the traits of specific colonizing species provide target ecosystem functions (and thus services) in real-world ecological systems compared to unaltered species assemblage requires empirical evidence. To achieve this, we suggest a research agenda at the interface of conservation, functional and ecosystem ecology, and an intensified societal/political discussion on conservation foci under an increasingly altered environment. Ultimately, the decision seems an economical one – if endless resources were available to conservation programs, nobody would ever argue to not preserve native and eradicate invasive species. However, given multiple global changes acting concurrently, and limited resources for conservation/restoration projects that have to service a large number of requirements (e.g., related to aesthetics, culture, carbon sequestration, and water quality), biodiversity – functional ecology trade-offs have to be considered carefully.

## Data Availability Statement

The original contributions presented in the study are included in the article/supplementary material, further inquiries can be directed to the corresponding authors.

## Author Contributions

SL and BR drafted the manuscript. SL provided the artwork. Both authors jointly revised and approved the manuscript.

## Conflict of Interest

The authors declare that the research was conducted in the absence of any commercial or financial relationships that could be construed as a potential conflict of interest.

## Publisher’s Note

All claims expressed in this article are solely those of the authors and do not necessarily represent those of their affiliated organizations, or those of the publisher, the editors and the reviewers. Any product that may be evaluated in this article, or claim that may be made by its manufacturer, is not guaranteed or endorsed by the publisher.
